# Supplementary material for: Development of chemokine network inhibitors using combinatorial saturation mutagenesis
Source: Commun Biol. 2025 Apr 3;8:549. doi: 10.1038/s42003-025-07778-6 (PMC11969024; doi:10.1038/s42003-025-07778-6)
Supplement: Supplementary file 4 — Supplementary Data 2 [file 42003_2025_7778_MOESM4_ESM.zip › SupplementaryData3/scripts/xcomb.tar!/./script/analysis/JK/HD2XC_heatmap.pdf]

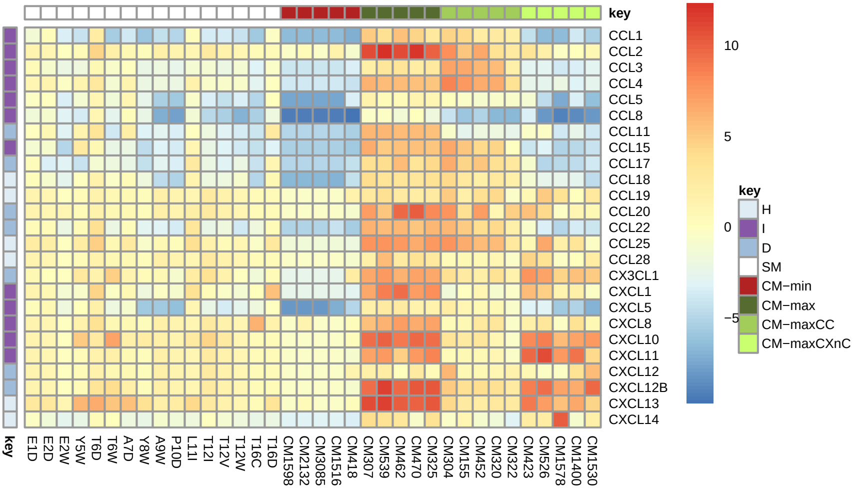
key
CCL1
CCL2
CCL3
CCL4
CCL5
CCL8
CCL11
CCL15
CCL17
CCL18
CCL19
CCL20
CCL22
CCL25
CCL28
CX3CL1
CXCL1
CXCL5
CXCL8
CXCL10
CXCL11
CXCL12
CXCL12B
CXCL13
CXCL14
key
E1D
E2D
E2W
Y5W
T6D
T6W
A7D
Y8W
A9W
P10D
L11I
T12I
T12V
T12W
T16C
T16D
CM1598
CM2132
CM3085
CM1516
CM418
CM307
CM539
CM462
CM470
CM325
CM304
CM155
CM452
CM320
CM322
CM423
CM526
CM1578
CM1400
CM1530
10
5
0
−5
key
H
I
D
SM
CM−min
CM−max
CM−maxCC
CM−maxCXnC
